# How do distinct facets of tree diversity and community assembly respond to environmental variables in the subtropical Atlantic Forest?

**DOI:** 10.1002/ece3.10321

**Published:** 2023-07-16

**Authors:** Joice Klipel, Rodrigo Scarton Bergamin, Marcus Vinicius Cianciaruso, Ana Carolina da Silva, Cristiane Follmann Jurinitz, João André Jarenkow, Kauane Maiara Bordin, Martin Molz, Pedro Higuchi, Rayana Caroline Picolotto, Vanderlei Júlio Debastiani, Sandra Cristina Müller

**Affiliations:** ^1^ Laboratório de Ecologia Vegetal (LEVEG), Programa de Pós‐Graduação em Ecologia, Departamento de Ecologia Universidade Federal do Rio Grande do Sul Porto Alegre Brazil; ^2^ School of Geography, Earth and Environmental Sciences University of Birmingham Birmingham UK; ^3^ Birmingham Institute of Forest Research (BIFoR) University of Birmingham Birmingham UK; ^4^ Departamento de Ecologia Universidade Federal de Goiás Goiânia Brazil; ^5^ Departamento de Engenharia Florestal, Centro de Ciências Agroveterinárias Universidade do Estado de Santa Catarina Lages Brazil; ^6^ Escola de Ciências da Saúde e da Vida Pontifícia Universidade Católica do Rio Grande do Sul (PUCRS) Porto Alegre Brazil; ^7^ Laboratório de Ecologia Vegetal e Fitogeografia, Departamento de Botânica Universidade Federal do Rio Grande do Sul Porto Alegre Brazil; ^8^ Museu de Ciências Naturais‐SEMA/RS Porto Alegre Brazil; ^9^ Laboratório de Ecologia Quantitativa, Programa de Pós‐Graduação em Ecologia, Departamento de Ecologia Universidade Federal do Rio Grande do Sul Porto Alegre Brazil

**Keywords:** altitude, biodiversity, community trait composition, functional traits, precipitation, soil variables, species richness

## Abstract

This study assessed the impact of altitude, precipitation, and soil conditions on species richness (SR), phylogenetic diversity (PD), and functional diversity (FD) standardized effect sizes in subtropical Brazilian Atlantic Forest tree communities. We considered specific trait information (FDs) for FD, reflecting recent adaptive evolution, contrasting with deeper phylogenetic constraints in FD. Three functional traits (leaf area‐LA, wood density‐WD, and seed mass‐SM) were examined for their response to these gradients. Generalized least squares models with environmental variables as predictors and diversity metrics as response variables were used, and a fourth‐corner correlation test explored trait‐environmental relationships. SR decreased with altitude, while PD increased, indicating niche convergence at higher altitudes. Leaf area and seed mass diversity also decreased with altitude. For LA, both FD and FDs were significant, reflecting filtering processes influenced by phylogenetic inheritance and recent trait evolution. For SM, only the specific trait structure responded to altitude. LA and SM showed significant trait‐environmental relationships, with smaller‐leaved and lighter‐seeded species dominant at higher altitudes. Soil gradients affect diversity. Fertile soils have a wider range of LA, indicating coexistence of species with different nutrient acquisition strategies. WD variation is lower for FDs. SM diversity has different relationships with soil fertility for FDs and FD, suggesting phylogeny influences trait variation. Soil pH influences WD and LA under acidic soils, with deeper phylogenetic constraints (FD). Environmental factors impact tree communities, with evidence of trait variation constraints driven by conditions and resources. Subtropical Atlantic forests' tree assemblies are mainly influenced by altitude, pH, and soil fertility, selecting fewer species and narrower trait spectra under specific conditions (e.g., higher altitudes, pH). Functional diversity patterns reflect both phylogenetic and recent evolution constraints, with varying strength across traits and conditions. These findings highlight the intricate processes shaping long‐lived species assembly across diverse environments in the Southern Brazilian Atlantic Forest.

## Graphical abstract

In this study, we measure different aspects of biodiversity in Subtropical Brazilian Atlantic Forest communities, taking into account the overlap between functional and phylogenetic structures. Our study shows that species richness, functional diversity, and phylogenetic diversity respond differently to environmental gradients, particularly when considering the impact of both the phylogenetic and specific structures on trait variation.

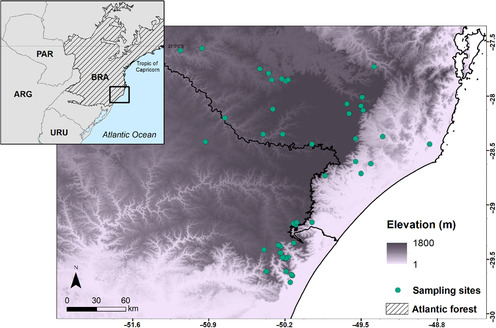

## 1 INTRODUCTION

Biodiversity encompasses various components, including taxonomic (species diversity), functional (differences in species traits), and phylogenetic diversity (diversity of species lineages) (Pavoine & Bonsall, 2011). Functional and phylogenetic diversity provide different yet complementary information about ecological and evolutionary differences among species, due to trait evolution (Webb et al., 2002). While several studies have examined multiple facets of biodiversity along environmental gradients (e.g., Dainese et al., 2015; de Bello et al., 2006; Luo et al., 2019), few have distinguished between the shared information among functional traits and phylogeny (de Bello et al., 2017). The shared information can be considerable, particularly when using phylogenetically conservative traits (de Bello et al., 2017; Diniz‐Filho et al., 2011), which makes understanding community assembly challenging across different regions and conditions. Frameworks that integrate species, functional, and phylogenetic diversity, while considering the overlapping and unique information from each component, may reveal new insights about species composition, ecological strategies, and adaptations across the evolutionary history of lineages in communities along environmental gradients. Such frameworks can help uncover the mechanisms driving biodiversity assembly.

Notably, functional traits provide information about species ecological differences (diversity) and habitat requirements (trait state) (HilleRisLambers et al., 2012), while phylogenetic diversity can allow us to understand the deeper evolutionary and biogeographical constraints on regional species distribution (de Bello et al., 2017; Gerhold et al., 2015). The ecological theory suggests that in addition to stochastic processes (Hubbell, 2001), deterministic processes, related to abiotic and biotic conditions, shape local communities by selecting species from the regional pool that can survive and persist in local conditions (Rapacciuolo & Blois, 2019; Vellend, 2010). Limiting similarity and environmental filtering are mechanisms that can act simultaneously along various environmental axes in a community during deterministic assembly (Cornwell & Ackerly, 2010; Gerhold et al., 2015; Weiher et al., 1998). In general, whereas limiting similarity is expected to exclude similar species in a community, environmental filtering is expected to select species with similar habitat requirements (HilleRisLambers et al., 2012; Kraft et al., 2007, 2015). However, convergence and divergence patterns in the functional and phylogenetic structures can arise from distinct processes, and these patterns can only be partially or not at all interrelated. Therefore, by separating the phylogenetic structure from the functional structure, it is potentially possible to differentiate the patterns and likely drivers of both the evolutionary and ecological components, including more recent adaptive evolution, within species assemblages (de Bello et al., 2017; Diniz‐Filho et al., 2011).

Altitudinal gradients provide insights into how environmental conditions drive community assembly and influence tree species, functional, and phylogenetic diversity (Ding, Shi, et al., 2019; Ding, Zang, et al., 2019; Pescador et al., 2015; Xu et al., 2017), as many variables change with altitude, such as temperature, cloudiness, atmospheric pressure, wind exposure, and area isolation. Precipitation is also a relevant factor influencing functional and phylogenetic diversity, where drier sites often limit diversity (Muscarella et al., 2016). Additionally, soil conditions like fertility, pH, and texture, along with climatic drivers, shape plant communities in terms of functional, phylogenetic, and taxonomic diversity (Bernard‐Verdier et al., 2013; Condit et al., 2013; Ordoñez et al., 2009). For instance, soil fertility in terms of cation exchange capacity can act as an environmental filter for nutrient‐poor tolerant species (Lambers et al., 2011; Quesada et al., 2012). Soil pH, on the other hand, can have a negative or positive effect on diversity in different floristic regions, depending on the prevailing pH conditions and the species pool that evolved to them (Pärtel, 2002). Soil texture (e.g., clay content and bulk density) is also relevant and often related to water retention, which is greater in clay‐rich soils, but it can further reduce nitrogen mineralization rates and affect plant‐available nutrients (Lambers et al., 2011; Quesada et al., 2012).

Overall, tree communities at higher altitudes experience harsh environmental conditions for plant establishment and growth that limit the number of coexisting species and clades (Bergamin et al., 2020). Species that are closely related and possess physiological and morphological traits allowing them to survive in these conditions are expected to exhibit phylogenetic clustering, with a reduction in trait dispersion and shifts in mean trait values along the altitudinal gradient (de Bello et al., 2013; Denelle et al., 2019). Convergence patterns in vegetative and regenerative traits of tree species (underdispersed traits), with resource conservation strategies such as smaller seeds (Qi et al., 2014; Wang et al., 2021), and lower leaf area and higher wood density (Ding, Shi, et al., 2019; Ding, Zang, et al., 2019; Kichenin et al., 2013), may arise due to environmental filtering or competitive hierarchy of specific trait values. In contrast, at lower altitudes with more favorable conditions, tree species may exhibit functional divergence (i.e., with overdispersed traits) and a prevalence of acquisitive resource strategies such as higher leaf area, lower wood density, and larger seeds (Díaz et al., 2015).

However, different climatic variables can affect each diversity component differently. Previous studies in mountain forests observed opposite trends for taxonomic and phylogenetic diversity with altitude: decreased species richness and increased phylogenetic diversity in higher altitudes (Culmsee & Leuschner, 2013). Evidence shows that phylogenetic diversity may increase with increasing altitude in tropical and subtropical altitudinal gradients (González‐Caro et al., 2014; Qian, 2017; Rezende et al., 2017), as some clades expanded their distributions to cooler regions where they have diversified slowly, resulting in species more distantly related to each other with similarities in adaptations to cold environments, following the niche convergence hypothesis (Qian & Ricklefs, 2016). Therefore, at high altitudes, increasing phylogenetic diversity of tree communities can be observed. In the same way, different traits may have contrasting patterns of trait dispersion along the gradient due to different assembly mechanisms and processes (e.g., selection or dispersal ability or possible lack of independence between the functional and phylogenetic structures) act simultaneously and may operate on different aspects of the organism's phenotype (Swenson, 2013). This means that one trait may be overdispersed while another is underdispersed. For example, along the same environmental gradient, seed mass can show patterns of divergence while leaf area convergence (Grime, 2006; Kraft et al., 2008), and this pattern can be phylogenetic dependent. Then, accounting for both overlapping, which includes the deeper phylogenetic constrains in trait variation, and unique information that reflects recent adaptive evolution independent of phylogenetic constraints (Diniz‐Filho et al., 2011; Gerhold et al., 2015) can be essential to unhide the multiple assembly processes that operate on the communities, as different traits may be affected by different mechanisms and processes (de Bello et al., 2017; Diniz‐Filho et al., 2011).

This study aims to assess shifts in biodiversity of tree communities across environmental gradients Southern Brazil's subtropical forests. The study considers species richness (SR), functional (ses.FD), and phylogenetic (ses.PD) diversity, as well as community trait composition. By analyzing three functional traits, phylogenetic relatedness, and community species abundance, the study aims to answer the questions: (i) to what extent are the four biodiversity measures similarly influenced by altitude, climate, and soil conditions? We hypothesized that restrictive conditions and resources lead to lower values of all diversity metrics due to filtering processes. However, different environmental variables may affect each diversity facet and trait differently. In this sense, SR and ses.FD of leaf area and wood density should be lower in higher altitudes, whereas ses.FD of seed mass and ses.PD should be higher. Under harsh conditions, communities are expected to have a predominance of trees with conservative strategies, such as small leaves, high wood density, and small seeds. (ii) Do the unique and overlapping information of traits and phylogeny on diversity measures respond similarly across distinct environmental gradients? The expectation is that analyzing FD from overlapping and unique information can uncover biodiversity assembly mechanisms and reveal whether species adaptations across environmental gradients follow a structured pattern in the evolutionary history of the lineages, regardless of the phylogeny.

## 2 METHODS

### 2.1 Community dataset

Our dataset consists of tree communities in the subtropical region of the Atlantic Forest biome in Brazil. The Atlantic Forest is a phytogeographical domain that encompasses various forest types and ecosystems, resulting in a heterogeneity of environments (Oliveira‐Filho & Fontes, 2000). The subtropical region is characterized by a mosaic of Araucaria Forest, natural grasslands (*Campos Sulinos*), moist coastal forests, and western semi‐deciduous forests, separated by plateaus (Oliveira‐Filho & Fontes, 2000). The climate is humid subtropical, with temperate summers and annual precipitation ranging between 1750 and 2500 mm (Alvares et al., 2013) without a dry season. The region experiences frost frequently during the winter (Backes, 2009).

**FIGURE 1:**
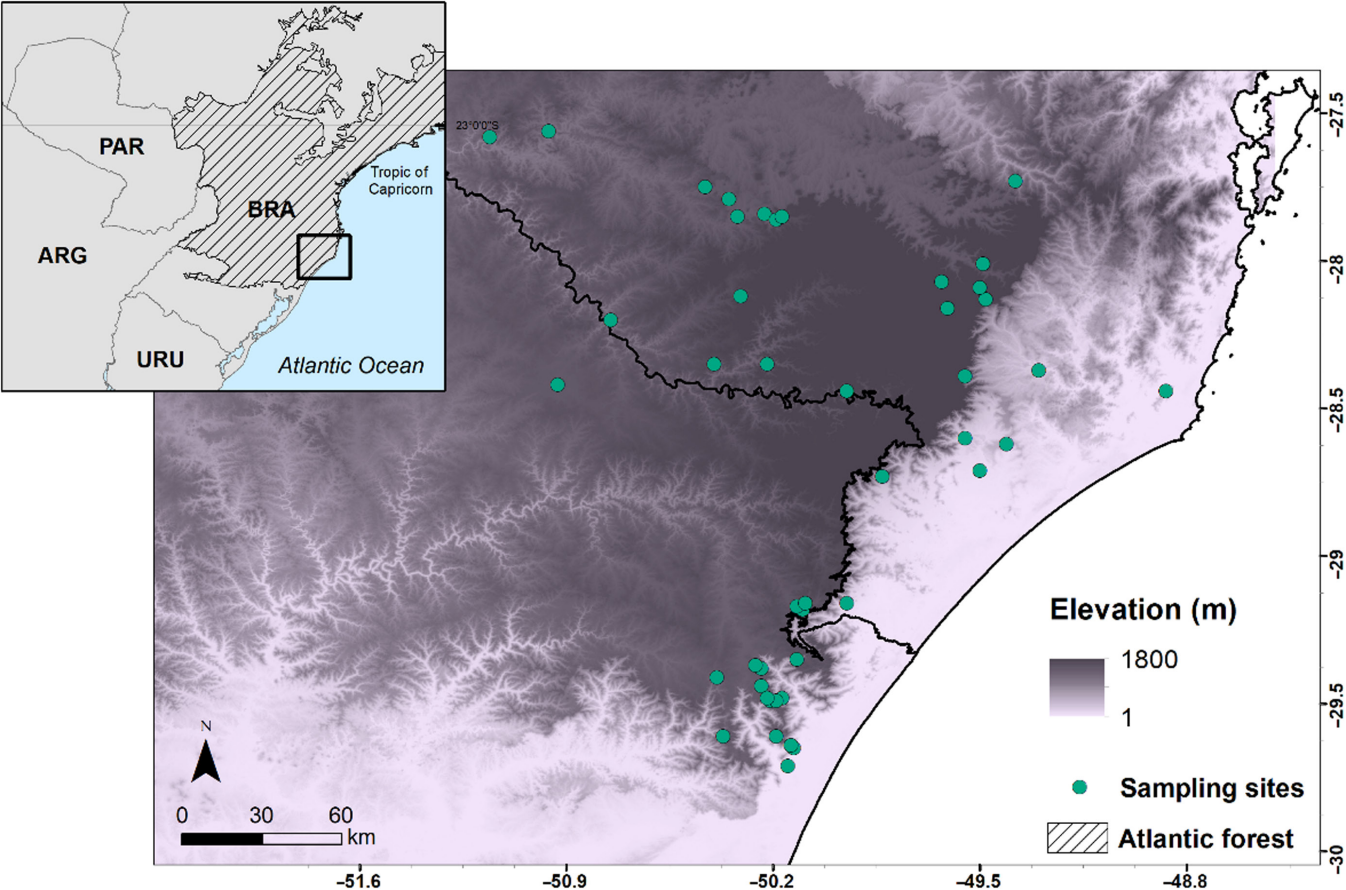
Location of the 50 tree community inventories along the subtropical Brazilian Atlantic Forest showing the distribution patterns of altitude (elevation, m) in the background.

We analyzed data from 50 tree community inventories in Southern Brazil, covering different altitudes from 40 to 1750 m (Figure 1). Plots situated at higher altitudes are predominantly covered by acid volcanic rocks, while plots at lower altitudes are characterized by predominantly basalt lithology in undulating mountainous terrain (Streck et al., 2008). These inventories were conducted by one of the authors of this study and included forests with varying sampling efforts (ranging from 1200 to 10,000 m^2^) but were all established in old‐growth forests (see Table S1 for details). We have standardized the tree inclusion threshold at stem diameter at breast height (DBH) >10 cm to ensure consistency across all inventories, including both angiosperms and gymnosperms, in each community. Even by using this threshold, our sample include small tree species from the families Myrtaceae, Melastomataceae, and Rubiaceae (e.g., *Calyptranthes conccina*, *Miconia cinerascens*, and *Psychotria suterella*), which contain characteristic elements of these subtropical forests. Moreover, the inventories encompassed representative environmental gradients in terms of altitude, precipitation, and soil types in the subtropical Atlantic Forest biome.

### 2.2 Environmental data

To investigate the effect of altitude and environmental gradients on diversity components and community trait composition, we obtained data on altitude value (m), climate, and soil variables. The climatic data included annual mean temperature (°C), temperature seasonality, minimum temperature of the coldest month (°C), mean annual precipitation (MAP, mm), and precipitation seasonality. These variables were extracted at 1 km of resolution from WorldClim (Fick & Hijmans, 2017). Altitude values were obtained from community locations. The soil variables (at 15 cm depth) were cation exchange capacity (CEC, cmolc/kg), as a fertility variable, clay content (g/kg), and bulk density (cg/cm^3^), characterizing soil texture and water retention capacity, soil pH, and soil nitrogen (cg/kg), related to the availability of nutrients. We obtained soil variables from SoilGrids (grids cells of 1 km of resolution) (Hengl et al., 2017).

### 2.3 Functional traits data

Plant functional traits used were leaf area (LA, cm^2^), wood density (WD, g.cm^3^), and dry seed mass (SM, g), which represent different plant strategy axes, including vegetative (LA), structural (WD), and reproductive (SM) features. These traits are based on the current understanding of the leaf economic spectrum (Wright et al., 2004), resource acquisition, dispersal and establishment rate (Kidson & Westoby, 2000), niche differentiation and persistence across different environmental conditions (Moles, 2018; Reich, 2014; Westoby et al., 2002). We were able to obtain LA and WD values to species level for up to 95% of the total species pool. For species values that were unavailable, we used genus mean values. We measured seed mass by collecting fruits from tree individuals in the study region and using bibliographic sources (e.g., Lorenzi, 2000; Seger et al., 2013; Sobral et al., 2006). We obtained SM values for 65% of the species and used the missForest algorithm (Debastiani et al., 2021; Penone et al., 2014) to estimate missing values, as seed mass is highly dependent on phylogenetic information (Moles et al., 2005). We also replaced missing data with average trait values from documented species and conducted further analysis to test if the associations between SM and environmental variables remained consistent using both methods of filling missing data (see Table S4). As the analysis revealed the same patterns using SM values from both methods of filling missing data, we used SM values obtained from the missForest algorithm. We gathered leaf area information from our Plant Ecology Lab database, available in the Try Plant Trait Database (Kattge et al., 2020). WD information was obtained from both regional (Missio et al., 2017; Oliveira et al., 2019) and global databases (Chave et al., 2009). Measurements and procedures to obtain LA and SM from species available in our database followed standardized protocols (Pérez‐Harguindeguy et al., 2013). There was no correlation among these functional traits (Table S2).

### 2.4 Phylogenetic tree

We used the “*S.PhyloMaker*” package to construct an ultrametric phylogeny for all 395 species in the community dataset, using the *PhytoPhylo megaphylogeny* as a backbone and adding genera or species absent from the megaphylogeny as basal polytomies within their families or genera (Qian & Jin, 2016). The PhytoPhylo megaphylogeny is a correction in the phylogeny for plant species from Zanne et al. (2014), which includes genetic data from GenBank and is the largest resolved phylogeny for plant species worldwide. An updated and expanded version of the phylogeny (i.e., PhytoPhylo) is time‐calibrated for all branches, includes all families of extant seed plants in the world. Our phylogenetic tree contains polytomies, or nodes with more than two descendent branches, which results in uncertainty about the correct branching order of taxa. To resolve polytomies, we used the function “*bifurcatr*” in package “PDcalc” (Rangel et al., 2015), which randomly resolves polytomies in a phylogenetic tree. We ran the algorithm 1000 times to explore the range of potential solutions and used all trees in the subsequent analyses.

### 2.5 Data analyses

We used the “*decouple*” approach (de Bello et al., 2017) to analyze the functional trait diversity of the species in our dataset and infer the unique and overlapping information of traits and phylogeny. This approach allows us to decouple phylogenetic diversity from functional diversity (the specific component of functional diversity; FDs) and calculate functional diversity accounting for evolutionary legacy (the phylogenetic component of functional diversity; FD) using multivariate analysis. To apply the “*decouple*” framework, we needed a Euclidean distance matrix for functional traits and the phylogenetic tree, both indicating the differences between species. The distance matrix for functional traits was obtained considering all traits separately (i.e., LA, WD, and SM). The functional traits were log‐transformed for normality purposes. To obtain trait information decoupled from phylogeny, the “decouple” function used phylogeny in the form of eigenvectors from a principal coordinate analysis as an explanatory variable and traits as response variables (Diniz‐Filho et al., 2011). The residuals of the model represent the variation in species' traits decoupled from phylogeny, which is computed as a distance matrix reflecting species trait distances independent of phylogeny.

We calculated functional (FD) and phylogenetic diversity (PD) using abundance‐weighted standardized effect sizes for mean pairwise dissimilarity (ses.MPD) (Pavoine et al., 2011; Webb, 2000; Weiher et al., 1998). For both ses.FD and ses.PD, negative values indicate lower diversity than expected by null model (independent swap, Kembel et al., 2010). Using 1000 phylogenetic trees, we computed functional diversity with overlapping information with phylogeny (ses.FD), specific functional diversity (ses.FDs), and phylogenetic diversity (ses.PD) for each community and each trait. We then calculated MPD for FD and PD 1000 times for each community and extracted the mean value of MPD for each community using the “mpd” function. Finally, we calculated rarefied species richness (using the “vegan” package and function “rarefy”) for species diversity, considering 465 individuals (See Table S1 for more details) (Oksanen et al., 2017).

To improve model estimation and reduce collinearity, we analyzed the correlation between predictor variables using Pearson's correlation. We observed a strong negative correlation between altitude and temperature variables (Table S3). As a result, we utilized altitude as a substitute for temperature variables, such as annual mean temperature, temperature seasonality, and minimum temperature of the coldest month. In addition, we found that temperature seasonality, minimum temperature of the coldest month, mean annual precipitation, and precipitation seasonality were highly correlated. Therefore, we only used mean annual precipitation. Among the noncorrelated soil variables, clay content, CEC, and soil pH were identified. However, we further used only CEC and pH because they best predicted diversity metrics (Figure S1).

To evaluate shifts in species richness, phylogenetic diversity, and functional diversity (ses.FD and ses.FDs) along altitudinal, precipitation (MAP), and soil (CEC and pH) gradients, we employed generalized least squares models. We included a covariate to account for differences in sampling effort and addressed spatial autocorrelation in the data. To determine the best autocorrelation structure, we compared models with various structures, such as Gaussian, spherical, exponential, and without autocorrelation structure, in a preliminary analysis. According to AICc, a Gaussian autocorrelation structure best fits the data. We used this structure for generating all models. To enable comparison of predictor variables within models, we standardized and centered all predictor variables to have zero mean and unit variance. The analyses were conducted in R version 3.6.3 Statistical Environment, using the “picante” (Kembel et al., 2010), “car” (Fox et al., 2014), and “nlme” (Pinheiro et al., 2016) packages.

Finally, the fourth‐corner approach was conducted to investigate the links between community trait composition and the environmental variables. This approach is considered robust for evaluating true correlations between community traits and environmental variables (Peres‐Neto et al., 2017; Zelený, 2018). The analysis involves a weighted correlation between community‐weighted trait means and weighted standardized environment, where the weights are total community abundances (Peres‐Neto et al., 2017). The fourth‐corner correlation analysis is conducted using max‐tests, which result in two independent permutations tests—rows or communities and columns or species. A conclusion is drawn that species sharing greater trait similarity exhibit more similar habitat affinities when row‐column permutation tests are significant (ter Braak et al., 2012). The R code provided in Peres‐Neto et al. (2017) was used for conducting the weighted correlation analyses.

## 3 RESULTS

**FIGURE 2:**
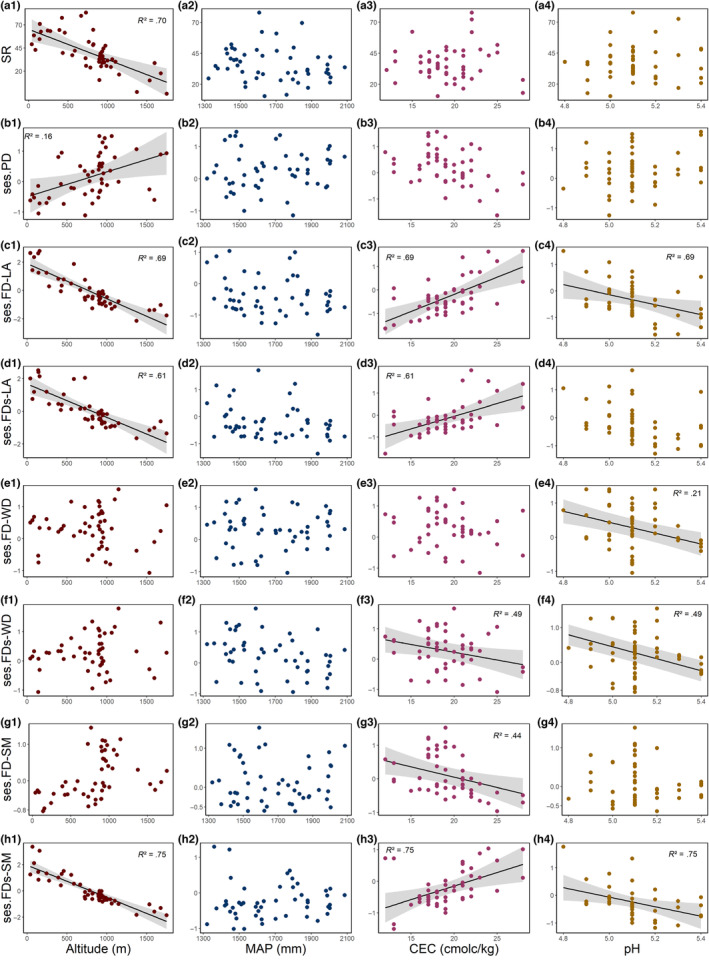
Relationships between species richness (SR; a1, a2, a3, a4), phylogenetic (ses.PD; b1, b2, b3, b4), and functional (ses.FDp and ses.FDs) diversity metrics, and elevation (red points), mean annual precipitation (blue points), cation exchange capacity (pink points) and clay content (yellow points) across 50 tree communities in subtropical Atlantic Forests in Southern Brazil. The functional traits considered are leaf area (ses.FD‐LA; c1, c2, c3, c4, and ses.FDs‐LA; d1, d2, d3, d4), wood density (ses.FD‐WD; e1, e2, e3, e4, and ses.FDs‐WD; f1, f2, f3, f4), and seed mass (ses.FD‐SM; g1, g2, g3, g4, and ses.FDs‐SM; h1, h2, h3, h4). Significant relationships (*P* < .05) have regression line fit.

In our study, we found that species richness (SR) was negatively associated with altitude (Figure 2a1), while phylogenetic diversity (ses.PD) was positively associated with altitude (Figure 2b1) (Table 1). Leaf area functional diversity, both with overlapping information of phylogeny (ses.FD‐LA) and with the specific component (ses.FDs‐LA), was negatively associated with altitude (Figure 2c1,d1) (Table 1) and positively associated with CEC (Figure 2c3,d3). Additionally, ses.FD‐LA was negatively associated with soil pH (Figure 2c4). The functional diversity of wood density (ses.FD‐WD) was negatively associated with soil pH (Figure 2e4). Similarly, FDs‐WD (i.e., the specific trait component) showed a negative relationship with CEC and soil pH (Figure 2f3,f4, respectively, Table 1). Seed mass diversity (ses.FD‐SM) was negatively associated with CEC (Figure 2g3), whereas ses.FDs‐SM was positively associated with CEC (Figure 2h3) and negatively associated with altitude and soil pH (Figure 2h1,h4). We also found that only species richness was positively correlated with sampling effort (Table 1), while precipitation did not appear to be relevant in the context of the region and community sets studied.

**TABLE 1:** Results for all generalized least square models tested to evaluate the influence of altitude, mean annual precipitation (MAP), cation exchange capacity (CEC), soil pH, and sampling effort (SE) on species richness, phylogenetic (ses.PD) and functional diversity (ses.FD) (LA = leaf area, WD = wood density, and SM = seed mass) in tree communities of subtropical Atlantic Forests in southern Brazil.

Response	*R* ^2^	Altitude		MAP		CEC		pH		SE	
		Coefficient	*P*	Coefficient	*P*	Coefficient	*P*	Coefficient	*P*	Coefficient	*P*
SR	.70	−0.59	<.001	‐	‐	‐	‐	‐	‐	0.40	<.001
ses.PD	.16	0.49	.02	‐	‐	‐	‐	‐	‐	‐	‐
ses.FD‐LA	.69	−1.03	<.001	‐	‐	0.52	<.001	−0.27	.02	‐	‐
ses.FDs‐LA	.61	−0.91	<.001	‐	‐	0.44	<.001	‐	‐	‐	‐
ses.FD‐WD	.21	‐	‐	‐	‐	‐	‐	−0.35	<.001	‐	‐
ses.FDs‐WD	.49	‐	‐	‐	‐	−0.23	.03	−0.34	<.001	‐	‐
ses.FD‐SM	.44	‐	‐	‐	‐	−0.27	.04	‐	‐	‐	‐
ses.FDs‐SM	.75	−1.08	<.001	‐	‐	0.32	.006	−0.26	.02	‐	‐

*Note*: The table shows the models with *R* ^2^, standardized coefficients, and *P*‐values for significant variables.

Abbreviations: ses.FD, nondecoupled functional diversity; ses.FDs, decoupled functional diversity; ses.PD, phylogenetic diversity; SR, rarefied species richness.

Our analysis using fourth‐corner correlations revealed significant (max‐test, *P* = .01) composite trait‐environmental relationships, specifically between leaf area (LA) and seed mass (SM) with the altitudinal gradient (Table 2). Also, LA was positively correlated with soil pH. We observed a shift in dominance along the altitudinal gradient, with larger‐leaved and heavier‐seeded species being replaced by those with smaller leaf area and lighter seeds. We observed a shift in leaf size dominance along a soil pH gradient, with larger leaves being dominant in more basic soils and smaller leaves in more acidic soils.

**TABLE 2:** Results for trait‐environmental relationships using fourth‐corner correlation.

	Altitude		MAP		CEC		pH	
	Correlation	*P*‐max	Correlation	*P*‐max	Correlation	*P*‐max	Correlation	*P*‐max
LA	−.45	**.01**	−.14	.17	−.03	.76	.30	**.01**
WD	.008	.91	−.04	.53	−.10	.22	−.03	.76
SM	−.22	**.04**	−.01	.88	−.07	.39	.17	.07

*Note*: Environmental variables are altitude, mean annual precipitation (MAP), cation exchange capacity (CEC), and soil pH. The functional traits are leaf area (LA), wood density (WD), and seed mass (SM). Max‐test considers the highest *P*‐value across species‐ and community‐level tests. In bold are the significant *P*‐values.

## 4 DISCUSSION

Studies investigating community assemblages across environmental gradients often use species richness, phylogenetic, and functional analyses, but they rarely evaluate these diversity facets together and often do not consider the possible overlap between functional and phylogenetic information. In this study, we demonstrated a variety of patterns of different diversity facets (species richness, phylogenetic and functional diversity), suggesting mixed processes drive tree assemblage patterns along the altitudinal gradient. Leaf area exhibited an underdispersion pattern with increasing altitude, with and without phylogenetic information, suggesting that the altitudinal gradient is a strong filter on recent diversification processes determining clustering patterns of leaf area size in tree species assemblages. Conversely, seed mass exhibited an underdispersion pattern with increasing altitude only when considering the specific functional diversity information. The specific functional diversity information evidence that tree community assembly is driven by environmental conditions in higher altitude areas (e.g., filtering process due to local harsher conditions) and/or by species interactions on the level of trait variability among species (e.g., effect of competitive hierarchy—presence and/or higher abundance of species with narrower trait values irrespective of species relatedness). In these areas, there is lower variability of leaf area and seed mass with a clear predominance of trees with small leaves and lighter seeds. Leaf area, wood density, and seed mass diversity were related to soil gradients, and the influence of phylogenetic information on these relationships varied.

### 4.1 Diversity patterns

Altitude strongly influences tree species richness and phylogenetic diversity, but they exhibit different tendencies. Species richness decreases with altitude due to environmental factors such as lower temperature, lower temperature seasonality, higher cloudiness, frost events, differences in land area, and isolation effects at higher altitudes, which constrain the distribution of tree species to higher altitudes. However, contrary to the findings of other studies in the Atlantic Forest (Bergamin et al., 2020; Giehl & Jarenkow, 2012; Mariano et al., 2020), we observed an increase in phylogenetic diversity with altitude, suggesting that niche convergence, rather than niche conservatism, plays a primary role in driving community assembly in the subtropical forests across the altitudinal gradient (Qian & Ricklefs, 2016). Despite most species originating in tropical environments under warmer and wetter conditions, a few species from some clades evolved ecological traits to persist in colder environments at higher altitudes, although adaptations to colder conditions may be difficult (Hawkins et al., 2011; Wiens & Donoghue, 2004). Then, we found a pattern of higher phylogenetic diversity under higher altitudes in the subtropical Atlantic Forest, which is consistent with the hypothesis that a few distantly related clades might have evolved the same ecological traits through niche convergence (Qian, 2017). This pattern emerged when controlling for species richness and comparing observed communities with randomly assembled composition (i.e., ses.PD), and considering the Gymnosperms, which are early diverging lineages known to thrive at higher altitudes. Moreover, the measurement of evolutionary diversity in communities involves different metrics and species pools that can yield different results. In our study, we employed the MPD approach, which calculates the distances across the phylogenetic tree by averaging pairwise distances of all species to quantify evolutionary diversity, and thus the presence of Gymnosperms surely influenced the results. In Bergamin et al. (2020) and Mariano et al. (2020), phylogenetic diversity was based on Angiosperms species only, and an opposite pattern with altitude was observed (i.e., a negative relation). By considering all tree species present in the communities of subtropical Atlantic forests along the altitudinal gradient, we had a broader understanding of evolutionary and biogeographic constraints of local assemblies.

A decrease in functional diversity along the altitudinal gradient was observed for leaf area with and without the phylogenetic information on the traits (FD and FDs) and seed mass, but only when we removed the evolutionary legacy on the trait (FDs). Our findings highlight that shift to lower diversity of leaf area toward higher elevations reflects an adaptation to climatic (i.e., lower temperatures and frost) and nonclimatic effects (i.e., land area and isolation effects) structured in both the phylogenetically inherited and recent components. Indeed, it is noteworthy that when the phylogenetic structure is removed from the trait (FDs), the resulting diversity patterns show little overlap of information. This suggests that the specific component, independent of phylogenetic constraints (i.e., deep evolutionary history), plays a crucial role in determining diversity values of leaf area along the altitudinal gradient. It indeed implies that the variation in this trait may be strongly influenced by more recent diversification processes. On the other hand, seed mass diversity across elevation gradients is independent of phylogeny, indicating that species have undergone recent trait differentiation to cope with colder environments (Webb et al., 2002). The clustered patterns observed in the diversity based on phylogeny, leaf area, and seed mass evidence that higher altitude conditions might be limiting and shaping tree community assemblies across altitudinal gradients in the Atlantic Forest, selecting similar species with a narrower range of ecological strategies in highland forests.

In fact, we observed species with similar functional traits adapted to harsh environmental conditions at higher altitude communities (Keddy, 1992), whereas at lower altitudes, in mild environmental conditions, we observed the co‐occurrence of phylogenetically close related species with a wider range of ecological strategies. This was characterized by higher functional diversity in terms of leaf area and seed mass. In this context, biotic pressures might impose limiting similarity processes with higher resource partitioning among coexisting species, resulting in higher functional diversity (Chesson, 2000). Additionally, past ecological conditions can help to elucidate the current diversity patterns in the subtropical Atlantic Forest (Carnaval et al., 2014; Costa et al., 2018; Duarte et al., 2014). Lower altitude regions are characterized by long‐term climate stability, resulting in higher species accumulation (i.e., higher richness) and functional diversity (Gerhold et al., 2018). In contrast, climatic fluctuations at higher altitudes probably drive lower species accumulation with species distantly related and lower functional diversity due to the neoendemisms and co‐occurrence of basal and younger clades (Bergamin et al., 2020; Gerhold et al., 2018; Massante et al., 2019). Thus, species that dispersed to higher altitudes after the last glacial period were those with similar leaf area, which was smaller compared to lowland forests. However, regarding the seed mass trait, it appears to have recently diversified, as the FD pattern was evident only without the influence of deep branches in phylogeny (Diniz‐Filho et al., 2011). These results emphasize that community assembly processes may diverge based on distinct functional traits.

Our study revealed significant and opposing relationships between functional diversity and soil variables, and the influence of phylogenetic information on these relationships varied. Specifically, we observed that in nutrient‐rich soil with a high CEC, tend to favor tree species with a wider range of leaf area for both historical and recent components. Fertile soil facilitates rapid nutrient acquisition, conserving resources in leaf tissues (Hodgson et al., 2011; Jager et al., 2015; Ordoñez et al., 2009) and promoting different coexistence strategies, ultimately resulting in higher leaf diversity. Contrary to our expectations, greater resource availability in our forest communities favors functional convergence in woody density, and recent adaptative component (FDs) contribute the most to the overall wood diversity pattern. To our knowledge, this result has not been previously reported. Further studies are necessary to better understand the relationships between soil conditions and woody diversity patterns in high‐diverse forests in tropical and subtropical regions.

Additionally, the diversity of wood density (FD and FDs) and leaf area (FD) were negatively associated with soil pH. Specifically, under more acidic soils (pH < 5), we observed a wider range of wood density and leaf area values, indicating functional divergence. This pattern aligns with the theory that local‐scale plant diversity and soil pH are linked to evolutionary history (Pärtel, 2002). In the Brazilian subtropical region, characterized by predominantly shallow and acidic soils, we observed more species adapted to those conditions in terms of leaf and structural traits, leading to higher diversity. This suggests that the unique soil properties in this region have contributed to the evolution of distinct leaf and wood density attributes. Notably, our results also indicated little overlap of information between wood density and phylogeny. This finding further supports the notion that the variation in wood density is influenced not only by deep evolutionary history but also by more recent diversification processes.

Interestingly, seed mass diversity exhibited opposite relationships with soil fertility depending on the component considered, either phylogenetic or specific. In nutrient‐poor soil, seed mass diversity tends to be higher due to the inherent phylogenetic component of the trait variation. In sites with limited resources, a broader range of reproductive strategies can coexist, and this relationship is dependent on older lineages that retain ancestral trait states (Diniz‐Filho et al., 2011). However, when considering seed mass diversity without phylogenetic information, we observed a wider range of reproductive strategies in nutrient‐rich soil, and the same was true for more acidic soils (pH < 5). Unexpectedly, pH was associated with the specific component. This suggests that specific trait variation allows co‐occurrence of species with different seed mass, and it is driven by recent diversification. Additionally, reproductive traits may exhibit different trends than foliar and structural traits with soil variables (Jager et al., 2015), and these relationships need to be further explored.

### 4.2 Trait composition patterns

We found significant shifts in leaf area and seed mass (LA, SM) along the altitudinal gradient in community trait composition, and LA was positive correlated with soil pH, which reflect different ecological strategies according to distinct environmental conditions (Díaz et al., 2015; Poorter et al., 2008). LA is associated with a light‐capture surface and is essential for photosynthesis and plant thermodynamics (Moles, 2018; Zhang et al., 2017) and often is associated with soil conditions (Ordoñez et al., 2009). Larger leaves can capture more light for photosynthesis but maintain lower thermal stability (more overheating and freezing) (Swenson et al., 2011). Under less stressful conditions, such as the lowland Atlantic forests, where species compete for light without having to maintain thermal stability, species with higher LA predominate in communities. Similarly, more alkaline soils have a predominance of larger leaves, which may be associated with increased nutrient availability (White, 2012). In contrast, species with more conservative strategies (lower LA) predominate under more stressful conditions, such as those observed at higher altitudes. The prevalence of lower LA values in tree communities at higher altitudes in subtropical Atlantic Forest reflects abiotic filters mediating the selection for smaller leaves to maintain thermal stability, since such communities experience lower temperatures and frost events, especially during the winter.

Moreover, we found that in tree communities at higher altitudes, there was a predominance of small seeds compared to those at lower altitudes. Seed mass is considered a trait highly phylogenetically dependent (Moles et al., 2005) since it reflects fundamental aspects of reproductive strategy (Moles et al., 2004). However, our study revealed a recent pattern of adaptation toward lighter seed mass in highland forests, independent of phylogenetic information (Figure 2e), indicating convergence in SM values toward higher altitude conditions. Past evolution and phylogenetic history determine seed mass (Wang et al., 2021), which affects the ability of lineages to colonize sites or persist under different environmental conditions. Since higher altitudes impose more stressful environments, such as lower temperatures, frost events, and lower resource availability to plants (e.g., lower radiation due to cloudiness), the conditions in highland forests may have selected species with lighter seed mass that produce more seeds in the same reproductive event, promoting higher dispersal opportunities (Cornelissen et al., 2003).

## 5 CONCLUSIONS

Our study highlights the importance of considering various diversity facets and traits to understand the impact of environmental gradients on community assembly. We found that different facets of tree diversity respond differently to environmental gradients, and the phylogenetic and specific trait component reveal different mechanisms behind forest patterns. In highland forests, a smaller pool (SR) of distantly related species (ses.PD) can establish with similar traits due to adaptations to environmental conditions. Moreover, in the Southern Brazilian Atlantic Forest, functional trait variation is unrelated to phylogenetic information, at least for leaf area and wood density, as there is limited overlap between the phylogeny and the specific components of these traits. Divergences or convergences in the functional structure were not associated with the pattern of phylogenetic information of traits. These variations may reflect processes that extend beyond evolutionary history and encompass factors within the recent environment. Regarding the diversity of seed mass, the relationships with environmental gradients were primarily influenced by recent trait adaptations, becoming evident only after removing the phylogenetic influence on the trait information. Our findings provide evidence of the complex processes that influence the assembly of long‐lived species over evolutionary timescales and across diverse environmental gradients, such as trees in the Southern Brazilian Atlantic Forest region.

## AUTHOR CONTRIBUTIONS

**Joice Klipel:** Conceptualization (lead); data curation (equal); formal analysis (lead); methodology (lead); writing – original draft (lead). **Rodrigo Scarton Bergamin:** Data curation (equal); supervision (equal); writing – review and editing (equal). **Marcus Vinicius Cianciaruso:** Writing – review and editing (equal). **Ana Carolina da Silva:** Data curation (equal); writing – review and editing (equal). **Cristiane Follmann Jurinitz:** Data curation (equal); writing – review and editing (equal). **João André Jarenkow:** Data curation (equal); writing – review and editing (equal). **Kauane Maiara Bordin:** Data curation (equal); writing – review and editing (equal). **Martin Molz:** Data curation (equal); writing – review and editing (equal). **Pedro Higuchi:** Data curation (equal); writing – review and editing (equal). **Rayana Caroline Picolotto:** Data curation (equal); writing – review and editing (equal). **Vanderlei Júlio Debastiani:** Formal analysis (supporting); writing – review and editing (equal). **Sandra Cristina Müller:** Supervision (lead); writing – review and editing (equal).

## Supporting information


Appendix S1

## Data Availability

The data used in this study are available in the supplementary information.
