# Supplementary material for: How do distinct facets of tree diversity and community assembly respond to environmental variables in the subtropical Atlantic Forest?
Source: Ecol Evol. 2023 Jul 16;13(7):e10321. doi: 10.1002/ece3.10321 (PMC10350641; doi:10.1002/ece3.10321)
Supplement: Supplementary file 1 — Appendix S1 [file ECE3-13-e10321-s001.docx]

**How do distinct facets of tree diversity and community assembly respond to environmental variables in the subtropical Atlantic Forest?**

Joice Klipel^1,*^, Rodrigo Scarton Bergamin^1,2,3^, Marcus Vinicius Cianciaruso^7^, Ana Carolina da Silva^4^, Cristiane Follmann Jurinitz^5^, João André Jarenkow^6^, Kauane Maiara Bordin^1^, Martin Molz^8^, Pedro Higuchi^4^, Rayana Caroline Picolotto^1^, Vanderlei Júlio Debastiani^9^, Sandra Cristina Müller^1^

^1^ Laboratório de Ecologia Vegetal (LEVEG), Programa de Pós-Graduação em Ecologia, Departamento de Ecologia, Universidade Federal do Rio Grande do Sul, Avenida Bento Gonçalves, 9500, Porto Alegre, RS

^2^ School of Geography, Earth and Environmental Sciences, University of Birmingham

^3^ Birmingham Institute of Forest Research (BIFoR), University of Birmigham

^4^ Centro de Ciências Agroveterinárias, Departamento de Engenharia Florestal, Universidade do Estado de Santa Catarina, Lages, SC

^5^ Escola de Ciências da Saúde e da Vida, Pontifícia Universidade Católica do Rio Grande do Sul (PUCRS), Avenida Ipiranga, 6681, Porto Alegre, RS

^6^ Laboratório de Ecologia Vegetal e Fitogeografia, Departamento de Botânica, Universidade Federal do Rio Grande do Sul, Avenida Bento Gonçalves, 9500, Porto Alegre, RS

^7^ Departamento de Ecologia, Universidade Federal de Goiás, Avenida Esperança, Goiânia, GO

^8^ Museu de Ciências Naturais- SEMA/RS, Rua Dr. Salvador França, 1427, Porto Alegre, RS

^9^ Laboratório de Ecologia Quantitativa, Programa de Pós-Graduação em Ecologia, Departamento de Ecologia, Universidade Federal do Rio Grande do Sul, Avenida Bento Gonçalves, 9500, Porto Alegre, RS

^*^ Corresponding author (E-mail: klipeljoice@gmail.com); https://orcid.org/0000-0003-3936-9692

**Supplementary Material**

**SM Table 1:** List of communities and their respective information concerning longitude (Long), latitude (Lat), sampling effort (m²), altitude, mean annual precipitation (MAP), cation exchange capacity (CEC), soil pH (*10), rarefied species richness (465 individuals considered),

phylogenetic (ses.PD) and functional diversity metrics (ses.FD and ses.FDs). Plant functional traits used were fresh leaf area (LA- cm²), wood density (WD- g.cm³), and seed mass (g). The diversity metrics measured to each community were functional diversity from overlapping components (FD), functional diversity from specific components (FDs), and phylogenetic diversity (PD). To run the analysis, we calculated FDs and PD using 1000 phylogenetic trees and we extracted the mean value of diversity metrics measured in each community to measure the standardized effect size (independent swap). The analyses were conducted in R version 3.6.3 Statistical Environment, using the ‘vegan’ (Oksanen et al., 2017) and ‘FD’ (Laliberté et al., 2015) packages.

| Long | Lat | Sampling Effort | Elevation | MAP | CEC | pH | Species Richness | ses.PD | ses.FD LA | ses.FDs LA | ses.FD WD | ses.FDs WD | ses.FD SM | ses.FDs SM |
| --- | --- | --- | --- | --- | --- | --- | --- | --- | --- | --- | --- | --- | --- | --- |
| -50.15 | -29.71 | 10000 | 88 | 1528 | 21 | 53 | 58.51 | -0.51 | 2.41 | 1.28 | 0.38 | -0.06 | -0.48 | 1.84 |
| -50.17 | -29.48 | 2100 | 819 | 1936 | 25 | 52 | 40.24 | -0.61 | -0.77 | -0.68 | 0.35 | 0.59 | -0.49 | -0.22 |
| -50.37 | -29.61 | 10000 | 824 | 2004 | 20 | 51 | 58.33 | 0.37 | -0.40 | -0.55 | 0.16 | -0.47 | -0.62 | -0.16 |
| -48.87 | -28.44 | 4000 | 143 | 1469 | 13 | 51 | 73.07 | -0.63 | 0.46 | 1.82 | -0.60 | 0.53 | -0.44 | 0.32 |
| -49.5 | -28.71 | 4000 | 41 | 1321 | 13 | 51 | 52.77 | -0.24 | 1.93 | 1.55 | 0.65 | 0.68 | 0.07 | 0.70 |
| -49.3 | -28.37 | 10000 | 157 | 1445 | 13 | 48 | 78.25 | -0.51 | 2.66 | 2.08 | 1.04 | 0.66 | -0.31 | 2.93 |
| -49.95 | -29.16 | 4000 | 244 | 1523 | 18 | 51 | 65.2 | -0.65 | 1.15 | 1.16 | 0.63 | 0.44 | -0.53 | 1.23 |
| -49.55 | -28.6 | 10000 | 140 | 1373 | 17 | 51 | 71.51 | -0.02 | 2.55 | 2.54 | -0.43 | -0.76 | -0.70 | 1.15 |
| -49.41 | -28.62 | 4000 | 68 | 1360 | 12 | 51 | 62 | 0.05 | 0.56 | 0.14 | 0.75 | 0.76 | 0.20 | 2.73 |
| -49.83 | -28.73 | 10000 | 728 | 1606 | 22 | 51 | 97.36 | -0.61 | 1.29 | 2.48 | 0.18 | -0.08 | -0.63 | 0.07 |
| -50.39 | -29.41 | 10000 | 913 | 2086 | 17 | 51 | 47.18 | 0.88 | -0.99 | -0.93 | 0.44 | 0.49 | 1.18 | -0.37 |
| -50.4 | -28.35 | 4000 | 988 | 1511 | 21 | 51 | 27.08 | 0.95 | -0.74 | -0.69 | -0.80 | -0.72 | 0.30 | -0.37 |
| -49.95 | -28.44 | 4000 | 1148 | 1591 | 20 | 52 | 16.98 | -0.01 | -0.80 | -0.73 | 1.36 | 1.60 | 1.06 | -1.09 |
| -50.22 | -28.35 | 4000 | 907 | 1484 | 18 | 51 | 35 | 1.54 | -0.99 | -0.48 | 1.16 | 1.23 | 0.89 | -0.83 |
| -49.49 | -28.01 | 4000 | 1045 | 1600 | 19 | 51 | 24.85 | 0.35 | -0.92 | -0.93 | -0.80 | -0.63 | 0.35 | -0.65 |
| -50.93 | -28.42 | 10000 | 899 | 1636 | 18 | 50 | 23.97 | 0.42 | 0.00 | 0.01 | 1.02 | 0.53 | -0.42 | -0.25 |
| -50.12 | -29.35 | 2000 | 785 | 1880 | 20 | 54 | 16.78 | 0.23 | -0.42 | -0.22 | -0.13 | -0.53 | -0.41 | -0.33 |
| -49.48 | -28.13 | 10000 | 1752 | 1824 | 28 | 49 | 6.65 | 0.17 | -0.09 | -0.11 | 1.21 | -0.05 | -0.26 | -0.81 |
| -49.55 | -28.39 | 10000 | 1374 | 1759 | 28 | 49 | 9.3 | 0.29 | -0.43 | -0.36 | -0.22 | 0.05 | -0.23 | -0.79 |
| -50.23 | -27.84 | 9600 | 896 | 1428 | 16 | 43 | 45.31 | -0.39 | -0.32 | -0.06 | 1.48 | 1.58 | -0.28 | -0.49 |
| -49.38 | -27.73 | 4000 | 821 | 1621 | 15 | 50 | 64.73 | -0.50 | -0.96 | -0.63 | -0.26 | -0.55 | -0.37 | -0.38 |
| -50.75 | -28.19 | 10000 | 730 | 1527 | 19 | 52 | 41.07 | -1.02 | -0.70 | -0.45 | 0.39 | 0.17 | -0.73 | -0.87 |
| -51.16 | -27.58 | 10000 | 765 | 1592 | 19 | 54 | 65.99 | 0.24 | -0.30 | -0.42 | -0.60 | -0.35 | 1.45 | -0.41 |
| -50.31 | -28.12 | 10000 | 1102 | 1499 | 18 | 47 | 45.36 | 0.80 | -0.93 | -0.72 | 1.48 | 1.47 | 0.80 | -0.79 |
| -50.43 | -27.75 | 10000 | 940 | 1464 | 22.97 | 48 | 51.82 | 0.09 | -1.04 | -0.78 | 0.05 | 0.34 | 0.07 | -0.71 |
| -50.32 | -27.85 | 10000 | 949 | 1420 | 19.37 | 48 | 60.75 | 1.43 | -0.15 | -0.25 | 0.92 | 0.70 | 1.22 | -0.75 |
| -49.63 | -28.07 | 10000 | 1536 | 1767 | 23 | 38 | 22.17 | -0.49 | -0.80 | -0.73 | -1.10 | -0.66 | -0.36 | -0.76 |
| -50.35 | -27.79 | 10000 | 935 | 1426 | 24 | 38 | 57.27 | 0.22 | -0.98 | -0.58 | 0.18 | 0.59 | 0.07 | -0.51 |
| -49.5 | -28.09 | 2000 | 1599 | 1799 | 23.5 | 44 | 21.87 | -1.11 | -0.44 | -0.33 | -0.12 | -0.86 | -0.60 | -0.83 |
| -49.61 | -28.16 | 2000 | 1678 | 1781 | 24 | 44 | 10.93 | 0.46 | -0.22 | -0.03 | 0.25 | 1.10 | -0.33 | -0.64 |
| -50.19 | -27.86 | 10000 | 975 | 1449 | 23.66 | 48 | 63.57 | 1.42 | -0.61 | -0.48 | 0.62 | 1.07 | 0.98 | -0.82 |
| -50.17 | -27.85 | 10000 | 1062 | 1478 | 17 | 48 | 48.06 | 1.49 | -1.04 | -0.67 | 0.75 | 1.51 | 1.02 | -0.91 |
| -50.96 | -27.55 | 9600 | 724 | 1627 | 26.42 | 45 | 46.88 | 0.38 | -0.42 | 0.24 | 1.06 | 1.08 | 1.00 | -0.54 |
| -49.98 | -29.36 | 10000 | 460 | 1453 | 16.7 | 51 | 78.2 | -0.65 | 0.69 | 1.03 | -0.07 | -0.43 | -0.56 | 1.06 |
| -50.24 | -29.44 | 1200 | 934 | 2006 | 17 | 51 | 18 | 0.71 | -0.66 | -0.49 | 0.90 | 0.36 | 0.69 | -0.10 |
| -50.24 | -29.38 | 1200 | 898 | 1995 | 18 | 51 | 21.99 | 0.98 | -0.77 | -0.59 | 0.42 | -0.03 | 0.97 | -0.43 |
| -50.26 | -29.36 | 1200 | 920 | 2003 | 17 | 50 | 14 | 1.09 | -0.58 | -0.48 | 0.99 | 0.34 | 0.73 | -0.44 |
| -50.18 | -29.62 | 1200 | 555 | 1795 | 21 | 52 | 28.9 | -0.26 | 0.45 | 0.07 | 0.92 | 0.37 | -0.39 | 1.29 |
| -50.19 | -29.61 | 1200 | 392 | 1704 | 23 | 54 | 57.79 | 0.74 | 0.74 | 0.40 | -0.40 | -0.44 | -0.47 | 0.90 |
| -50.19 | -29.54 | 1200 | 588 | 1810 | 21 | 58 | 34 | -0.11 | 1.45 | 1.62 | -0.47 | -0.62 | -0.22 | 0.28 |
| -50.18 | -29.55 | 1200 | 434 | 1726 | 21 | 58 | 32.55 | 0.97 | 0.12 | 0.21 | -0.28 | -0.21 | -0.29 | 1.10 |
| -50.1 | -29.17 | 1200 | 947 | 1876 | 26.64 | 40 | 22.91 | 0.48 | -0.82 | -0.63 | 0.13 | -0.35 | 0.71 | -0.58 |
| -50.12 | -29.16 | 1200 | 959 | 1881 | 26.95 | 41 | 19.98 | 0.46 | -0.71 | -0.62 | 0.06 | -0.12 | 0.66 | -0.54 |
| -50.09 | -29.15 | 1200 | 1003 | 1877 | 30.38 | 38 | 22 | 0.00 | -0.78 | -0.49 | -0.32 | -0.55 | -0.13 | -0.70 |
| -50.21 | -29.48 | 1200 | 919 | 1989 | 19 | 51 | 28.83 | -0.23 | -0.78 | -0.74 | 0.51 | -0.47 | -0.08 | -0.08 |
| -50.19 | -29.48 | 1200 | 909 | 1982 | 19 | 52 | 22.95 | -0.20 | -0.58 | -0.47 | 0.84 | 0.10 | -0.32 | -0.09 |
| -50.22 | -29.48 | 1200 | 904 | 1990 | 18 | 49 | 24.97 | 0.50 | -0.70 | -0.61 | 0.01 | -0.39 | 0.40 | -0.30 |
| -50.14 | -29.64 | 2400 | 667 | 1846 | 22.33 | 57 | 73.85 | 0.48 | -0.06 | 0.04 | -0.17 | -0.24 | -0.61 | 0.45 |
| -50.13 | -29.64 | 2400 | 277 | 1624 | 24.4 | 50 | 60.09 | -0.55 | 0.17 | 0.37 | -0.07 | -0.16 | -0.52 | 0.63 |
| -50.14 | -29.64 | 2400 | 424 | 1702 | 32.43 | 43 | 73.19 | -0.22 | 0.13 | 0.22 | 0.04 | -0.05 | -0.60 | 0.54 |

**SM Table 2:** Correlation between the species functional traits used in this study. Plant functional traits measured were fresh leaf area (LA, cm²), wood density (WD, g.cm³), and seed mass (g). Correlation values above 0.7 were considered correlated (p < 0.05).

|  | LA | WD | SM |
| --- | --- | --- | --- |
| LA | 1 |  |  |
| WD | -0.13 | 1 |  |
| SM | -0.04 | 0.02 | 1 |

**SM Table 3**: Correlation between the variables used in this study: altitude, annual mean temperature (MAT), temperature seasonality (TS), minimum temperature of coldest month (MT), mean annual precipitation (MAP), precipitation seasonality (PS), clay content, cation exchange capacity (CEC), soil pH, bulk density, and soil nitrogen (N). Variables with correlation values above 0.7 were considered correlated.

|  | Altitude | MAT | TS | MT | MAP | PS | Clay | CEC | Ph | Bulk Density | N |
| --- | --- | --- | --- | --- | --- | --- | --- | --- | --- | --- | --- |
| Altitude | 1 |  |  |  |  |  |  |  |  |  |  |
| MAT | -0.98 | 1 |  |  |  |  |  |  |  |  |  |
| TS | -0.73 | 0.77 | 1 |  |  |  |  |  |  |  |  |
| MT | -0.95 | 0.91 | 0.55 | 1 |  |  |  |  |  |  |  |
| MAP | 0.38 | -0.52 | -0.51 | -0.26 | 1 |  |  |  |  |  |  |
| PS | -0.3 | 0.4 | 0.22 | 0.15 | -0.74 | 1 |  |  |  |  |  |
| Clay Content | 0.57 | -0.54 | -0.44 | -0.46 | 0.23 | -0.36 | 1 |  |  |  |  |
| CEC | 0.52 | -0.58 | -0.6 | -0.34 | 0.4 | -0.42 | 0.4 | 1 |  |  |  |
| pH | -0.37 | 0.3 | 0.18 | 0.5 | 0.14 | -0.38 | -0.25 | 0.19 | 1 |  |  |
| Bulk Density | -0.82 | 0.87 | 0.68 | 0.72 | -0.64 | 0.54 | -0.49 | -0.57 | 0.16 | 1 |  |
| N | 0.63 | -0.74 | -0.57 | -0.57 | 0.88 | -0.67 | 0.34 | 0.46 | -0.06 | -0.77 | 1 |

| **(a)** | **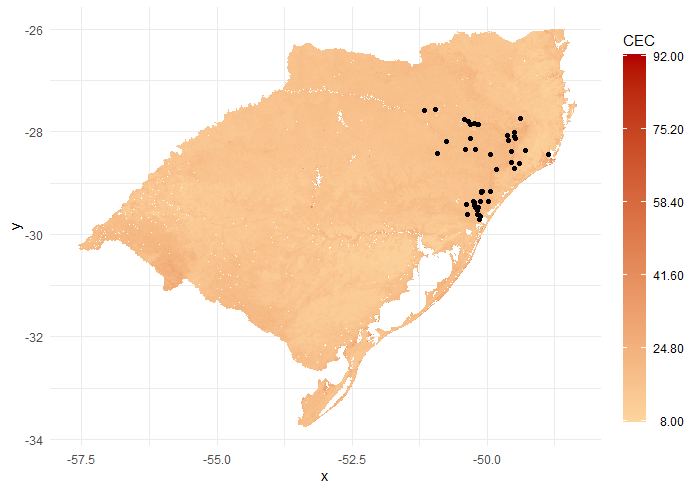** |
| --- | --- |
| **(b)** | **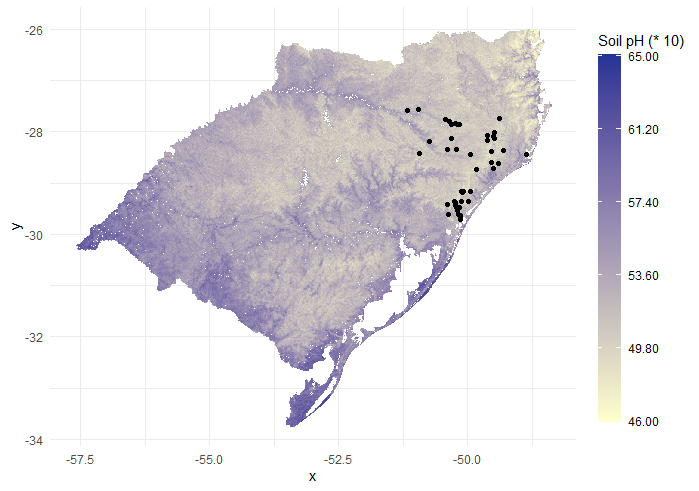** |

|  |  |
| --- | --- |
|  |  |

**SM Figure 1:** Location of the 50 tree communities’ surveys along the subtropical Brazilian Atlantic Forest showing the distribution patterns of cation exchange capacity (a) and soil pH (b) in the background.

**SM Table 4:** Seed mass values documented (SM- raw data) for the species from our study area, and SM values using two methods of filling missing data: missForest algorithm and average trait value of the species with SM values documented. Our analysis showed the same patterns using SM values from both methods.

| Species | SM (raw data) | SM (missForest algorithm ) | SM (average trait value) |
| --- | --- | --- | --- |
| Abarema langsdorffii | NA | 0.304609179 | 0.260589 |
| Acca sellowiana | 0.004629496 | 0.004629496 | 0.004629496 |
| Actinostemon concolor | 0.38 | 0.38 | 0.38 |
| Aegiphila brachiata | NA | 0.121312611 | 0.260589 |
| Aegiphila integrifolia | 0.0263 | 0.0263 | 0.0263 |
| Aegiphila sellowiana | NA | 0.135411096 | 0.260589 |
| Aiouea acarodomatifera | NA | 0.602195444 | 0.260589 |
| Aiouea pseudoglaziovii | 0.41369 | 0.41369 | 0.41369 |
| Aiouea saligna | NA | 0.654089808 | 0.260589 |
| Albizia edwallii | NA | 0.286558833 | 0.260589 |
| Albizia niopoides | NA | 0.255948389 | 0.260589 |
| Alchornea glandulosa | 0.05 | 0.05 | 0.05 |
| Alchornea triplinervia | 0.021111111 | 0.021111111 | 0.021111111 |
| Allophylus edulis | 0.047277778 | 0.047277778 | 0.047277778 |
| Allophylus guaraniticus | NA | 0.123760766 | 0.260589 |
| Allophylus petiolulatus | 0.07 | 0.07 | 0.07 |
| Alseis floribunda | 0.0003 | 3.00E-04 | 0.0003 |
| Alsophila setosa | NA | NA | 0.260589 |
| Amaioua guianensis | 0.008 | 0.008 | 0.008 |
| Aniba firmula | 2.17 | 2.17 | 2.17 |
| Annona cacans | 0.238948626 | 0.238948626 | 0.238948626 |
| Annona neosalicifolia | 0.43405 | 0.43405 | 0.43405 |
| Annona neosericea | 0.05 | 0.05 | 0.05 |
| Annona rugulosa | 0.46255 | 0.46255 | 0.46255 |
| Annona sylvatica | 0.2515 | 0.2515 | 0.2515 |
| Araucaria angustifolia | 5.487804878 | 5.487804878 | 5.487804878 |
| Aspidosperma australe | 0.2 | 0.2 | 0.2 |
| Aspidosperma olivaceum | 0.2 | 0.2 | 0.2 |
| Aspidosperma parvifolium | 0.2 | 0.2 | 0.2 |
| Aspidosperma tomentosum | 0.47 | 0.47 | 0.47 |
| Azara uruguayensis | NA | 0.1371198 | 0.260589 |
| Baccharis oblongifolia | NA | 0.127708002 | 0.260589 |
| Baccharis semiserrata | NA | 0.21348422 | 0.260589 |
| Banara parviflora | NA | 0.10168815 | 0.260589 |
| Banara tomentosa | 0.00083 | 0.00083 | 0.00083 |
| Bathysa australis | 0.00033264 | 0.00033264 | 0.00033264 |
| Bauhinia forficata | 0.08 | 0.08 | 0.08 |
| Blepharocalyx salicifolius | 0.015946297 | 0.015946297 | 0.015946297 |
| Boehmeria caudata | 0.0007 | 7.00E-04 | 0.0007 |
| Brosimum glaziovii | 0.263157895 | 0.263157895 | 0.263157895 |
| Brosimum lactescens | NA | 0.196686333 | 0.260589 |
| Buchenavia kleinii | NA | 0.397781899 | 0.260589 |
| Butia eriospatha | NA | 1.722619736 | 0.260589 |
| Byrsonima ligustrifolia | 0.959230769 | 0.959230769 | 0.959230769 |
| Byrsonima niedenzuiana | 0.1789 | 0.1789 | 0.1789 |
| Cabralea canjerana | 0.4992 | 0.4992 | 0.4992 |
| Calyptranthes concinna | 0.03795 | 0.03795 | 0.03795 |
| Calyptranthes grandifolia | 0.095238095 | 0.095238095 | 0.095238095 |
| Calyptranthes lucida | 0.0728 | 0.0728 | 0.0728 |
| Campomanesia guaviroba | 0.1351 | 0.1351 | 0.1351 |
| Campomanesia guazumifolia | 0.037727273 | 0.037727273 | 0.037727273 |
| Campomanesia rhombea | 0.00945 | 0.00945 | 0.00945 |
| Campomanesia xanthocarpa | 0.053431818 | 0.053431818 | 0.053431818 |
| Casearia decandra | 0.0162 | 0.0162 | 0.0162 |
| Casearia obliqua | 0.38 | 0.38 | 0.38 |
| Casearia sylvestris | 0.011904762 | 0.011904762 | 0.011904762 |
| Cecropia glaziovii | 0.000021739 | 2.17E-05 | 0.000021739 |
| Cedrela fissilis | 0.027964988 | 0.027964988 | 0.027964988 |
| Celtis iguanaea | 0.134860715 | 0.134860715 | 0.134860715 |
| Cestrum bracteatum | NA | 0.073987075 | 0.260589 |
| Cestrum intermedium | 0.0115 | 0.0115 | 0.0115 |
| Chionanthus filiformis | NA | 0.143840568 | 0.260589 |
| Chionanthus micranthus | NA | 0.13821708 | 0.260589 |
| Chionanthus trichotomus | NA | 0.120840551 | 0.260589 |
| Chrysophyllum gonocarpum | 0.2513 | 0.2513 | 0.2513 |
| Chrysophyllum inornatum | 0.19 | 0.19 | 0.19 |
| Chrysophyllum marginatum | 0.181818182 | 0.181818182 | 0.181818182 |
| Chrysophyllum viride | 0.524666667 | 0.524666667 | 0.524666667 |
| Cinnamodendron dinisii | 0.3571 | 0.3571 | 0.3571 |
| Cinnamomum amoenum | NA | 0.434838428 | 0.260589 |
| Cinnamomum glaziovii | 0.161290323 | 0.161290323 | 0.161290323 |
| Cinnamomum pseudoglaziovii | NA | 0.67981567 | 0.260589 |
| Citharexylum montevidense | 0.0526 | 0.0526 | 0.0526 |
| Citharexylum myrianthum | 0.42185 | 0.42185 | 0.42185 |
| Citharexylum solanaceum | 0.484518889 | 0.484518889 | 0.484518889 |
| Citronella gongonha | 0.2057 | 0.2057 | 0.2057 |
| Citronella paniculata | 0.666666667 | 0.666666667 | 0.666666667 |
| Clethra scabra | 0.00025 | 0.00025 | 0.00025 |
| Clusia criuva | 0.241 | 0.241 | 0.241 |
| Copaifera trapezifolia | 1.626 | 1.626 | 1.626 |
| Cordia americana | 0.17375 | 0.17375 | 0.17375 |
| Cordia ecalyculata | 0.185185185 | 0.185185185 | 0.185185185 |
| Cordia silvestris | 0.065232692 | 0.065232692 | 0.065232692 |
| Cordia trichotoma | 0.028409091 | 0.028409091 | 0.028409091 |
| Cordiera concolor | NA | 0.156705763 | 0.260589 |
| Cordyline spectabilis | NA | 0.442474687 | 0.260589 |
| Coussapoa microcarpa | 0.0006 | 6.00E-04 | 0.0006 |
| Coussarea contracta | NA | 0.090457224 | 0.260589 |
| Coutarea hexandra | 0.0003 | 3.00E-04 | 0.0003 |
| Crinodendron brasiliense | NA | 0.280474124 | 0.260589 |
| Croton macrobothrys | NA | 0.120422732 | 0.260589 |
| Cryptocarya aschersoniana | 1.337994445 | 1.337994445 | 1.337994445 |
| Cryptocarya mandioccana | NA | 0.893568208 | 0.260589 |
| Cryptocarya moschata | NA | 0.791599079 | 0.260589 |
| Cupania vernalis | 0.1573 | 0.1573 | 0.1573 |
| Cyathea atrovirens | NA | NA | 0.260589 |
| Cyathea corcovadensis | NA | NA | 0.260589 |
| Cyathea delgadii | NA | NA | 0.260589 |
| Cyathea phalerata | NA | NA | 0.260589 |
| Cybistax antisyphilitica | 0.036 | 0.036 | 0.036 |
| Dalbergia frutescens | NA | 0.261616345 | 0.260589 |
| Daphnopsis fasciculata | 0.03805 | 0.03805 | 0.03805 |
| Dasyphyllum brasiliense | 0.0005 | 5.00E-04 | 0.0005 |
| Dasyphyllum spinescens | 0.0005 | 5.00E-04 | 0.0005 |
| Dicksonia sellowiana | NA | NA | 0.260589 |
| Diospyros inconstans | 0.249865217 | 0.249865217 | 0.249865217 |
| Drimys angustifolia | 0.003897297 | 0.003897297 | 0.003897297 |
| Drimys brasiliensis | 0.0031 | 0.0031 | 0.0031 |
| Duguetia lanceolata | 0.625 | 0.625 | 0.625 |
| Duranta vestita | 0.0198 | 0.0198 | 0.0198 |
| Endlicheria paniculata | 0.67275 | 0.67275 | 0.67275 |
| Enterolobium contortisiliquum | 0.1802 | 0.1802 | 0.1802 |
| Erythrina falcata | 0.6452 | 0.6452 | 0.6452 |
| Erythroxylum cuneifolium | 0.01445 | 0.01445 | 0.01445 |
| Erythroxylum deciduum | 0.05 | 0.05 | 0.05 |
| Escallonia bifida | 0.0005 | 5.00E-04 | 0.0005 |
| Esenbeckia grandiflora | NA | 0.09359734 | 0.260589 |
| Eugenia bacopari | NA | 0.238868266 | 0.260589 |
| Eugenia beaurepairiana | NA | 0.579949527 | 0.260589 |
| Eugenia brasiliensis | NA | 0.371408094 | 0.260589 |
| Eugenia brevistyla | NA | 0.379321401 | 0.260589 |
| Eugenia burkartiana | 0.045 | 0.045 | 0.045 |
| Eugenia chlorophylla | NA | 0.589862179 | 0.260589 |
| Eugenia handroana | NA | 0.424214691 | 0.260589 |
| Eugenia handroi | 0.4 | 0.4 | 0.4 |
| Eugenia involucrata | 0.155161539 | 0.155161539 | 0.155161539 |
| Eugenia multicostata | 0.4574 | 0.4574 | 0.4574 |
| Eugenia neoverrucosa | NA | 0.280831739 | 0.260589 |
| Eugenia oeidocarpa | 0.6615 | 0.6615 | 0.6615 |
| Eugenia paracatuana | NA | 0.763702839 | 0.260589 |
| Eugenia platysema | NA | 0.838551332 | 0.260589 |
| Eugenia pluriflora | NA | 0.214900723 | 0.260589 |
| Eugenia pruinosa | 5.2379 | 5.2379 | 5.2379 |
| Eugenia pyriformis | 0.8547 | 0.8547 | 0.8547 |
| Eugenia ramboi | 0.1923 | 0.1923 | 0.1923 |
| Eugenia rostrifolia | 0.13 | 0.13 | 0.13 |
| Eugenia stigmatosa | NA | 0.496421102 | 0.260589 |
| Eugenia subterminalis | 0.133448276 | 0.133448276 | 0.133448276 |
| Eugenia ternatifolia | NA | 0.572260051 | 0.260589 |
| Eugenia uniflora | NA | 0.406849169 | 0.260589 |
| Eugenia uruguayensis | 0.28063125 | 0.28063125 | 0.28063125 |
| Eugenia verticillata | NA | 0.283714021 | 0.260589 |
| Euterpe edulis | 0.6504 | 0.6504 | 0.6504 |
| Faramea montevidensis | 0.05885 | 0.05885 | 0.05885 |
| Ficus adhatodifolia | 0.00121935 | 0.00121935 | 0.00121935 |
| Ficus cestrifolia | 0.0006 | 6.00E-04 | 0.0006 |
| Ficus eximia | NA | 0.085034664 | 0.260589 |
| Ficus luschnathiana | 0.0002 | 2.00E-04 | 0.0002 |
| Garcinia gardneriana | 2.9 | 2.9 | 2.9 |
| Gochnatia polymorpha | 0.005 | 0.005 | 0.005 |
| Guapira opposita | 0.0725 | 0.0725 | 0.0725 |
| Guarea macrophylla | 0.187966667 | 0.187966667 | 0.187966667 |
| Guatteria australis | NA | 0.43262741 | 0.260589 |
| Guazuma ulmifolia | 0.0063 | 0.0063 | 0.0063 |
| Gymnanthes klotzschiana | NA | 0.276264695 | 0.260589 |
| Handroanthus albus | 0.0117 | 0.0117 | 0.0117 |
| Handroanthus chrysotrichus | 0.0107 | 0.0107 | 0.0107 |
| Handroanthus pulcherrimus | NA | 0.063061246 | 0.260589 |
| Handroanthus umbellatus | 0.0186 | 0.0186 | 0.0186 |
| Heisteria silvianii | 0.5128 | 0.5128 | 0.5128 |
| Helietta apiculata | 0.0177 | 0.0177 | 0.0177 |
| Hennecartia omphalandra | 0.494 | 0.494 | 0.494 |
| Hieronyma alchorneoides | 0.0208 | 0.0208 | 0.0208 |
| Hirtella hebeclada | 0.51282051 | 0.51282051 | 0.51282051 |
| Ilex brevicuspis | 0.0020125 | 0.0020125 | 0.0020125 |
| Ilex dumosa | 0.00313529 | 0.00313529 | 0.00313529 |
| Ilex microdonta | 0.002608163 | 0.002608163 | 0.002608163 |
| Ilex paraguariensis | 0.007657143 | 0.007657143 | 0.007657143 |
| Ilex taubertiana | NA | 0.058494405 | 0.260589 |
| Ilex theezans | 0.0019 | 0.0019 | 0.0019 |
| Inga | 0.24 | 0.24 | 0.24 |
| Inga lentiscifolia | NA | 0.265999057 | 0.260589 |
| Inga marginata | 0.250875 | 0.250875 | 0.250875 |
| Inga sessilis | NA | 0.252234264 | 0.260589 |
| Inga striata | 0.251669912 | 0.251669912 | 0.251669912 |
| Inga virescens | 0.2382 | 0.2382 | 0.2382 |
| Jacaranda micrantha | 0.008 | 0.008 | 0.008 |
| Jacaranda puberula | 0.00496902 | 0.00496902 | 0.00496902 |
| Jacaratia spinosa | 0.0081 | 0.0081 | 0.0081 |
| Kaunia rufescens | NA | 0.148163098 | 0.260589 |
| Lamanonia ternata | 0.0007 | 7.00E-04 | 0.0007 |
| Laplacea acutifolia | 0.0034 | 0.0034 | 0.0034 |
| Leandra dasytricha | NA | 0.176779761 | 0.260589 |
| Leandra salicina | NA | 0.164352117 | 0.260589 |
| Licaria armeniaca | NA | 1.213348556 | 0.260589 |
| Lithraea brasiliensis | 0.0392 | 0.0392 | 0.0392 |
| Lonchocarpus campestris | NA | 0.209693544 | 0.260589 |
| Lonchocarpus cultratus | 0.1639 | 0.1639 | 0.1639 |
| Lonchocarpus grazielae | NA | 0.213917304 | 0.260589 |
| Luehea divaricata | 0.002733333 | 0.002733333 | 0.002733333 |
| Machaerium paraguariense | 0.3517 | 0.3517 | 0.3517 |
| Machaerium stipitatum | 0.0915 | 0.0915 | 0.0915 |
| Magnolia ovata | 0.19635 | 0.19635 | 0.19635 |
| Margaritaria nobilis | NA | 0.438123976 | 0.260589 |
| Marlierea excoriata | NA | 0.275046247 | 0.260589 |
| Marlierea sylvatica | NA | 0.304175418 | 0.260589 |
| Matayba elaeagnoides | 0.145949333 | 0.145949333 | 0.145949333 |
| Matayba intermedia | 1.20714286 | 1.20714286 | 1.20714286 |
| Maytenus | 0.02 | 0.02 | 0.02 |
| Maytenus aquifolia | 0.1505 | 0.1505 | 0.1505 |
| Maytenus boaria | NA | 0.084611826 | 0.260589 |
| Maytenus dasyclada | NA | 0.108602329 | 0.260589 |
| Maytenus evonymoides | 0.023302778 | 0.023302778 | 0.023302778 |
| Maytenus ilicifolia | 0.0308 | 0.0308 | 0.0308 |
| Maytenus schumanniana | NA | 0.106028305 | 0.260589 |
| Meliosma sellowii | 0.8315 | 0.8315 | 0.8315 |
| Meliosma sinuata | NA | 0.444768702 | 0.260589 |
| Miconia cabussu | 0.0013 | 0.0013 | 0.0013 |
| Miconia cinerascens | 0.001843262 | 0.001843262 | 0.001843262 |
| Miconia cinnamomifolia | 0.0004 | 4.00E-04 | 0.0004 |
| Miconia cubatanensis | NA | 0.111472401 | 0.260589 |
| Miconia eichleri | NA | 0.129203389 | 0.260589 |
| Miconia latecrenata | NA | 0.097769032 | 0.260589 |
| Miconia pusilliflora | NA | 0.07947317 | 0.260589 |
| Mimosa bimucronata | 0.0112 | 0.0112 | 0.0112 |
| Mimosa scabrella | 0.0182 | 0.0182 | 0.0182 |
| Mollinedia | 0.06 | 0.06 | 0.06 |
| Mollinedia fruticulosa | 0.05 | 0.05 | 0.05 |
| Mollinedia schottiana | 0.084 | 0.084 | 0.084 |
| Mollinedia triflora | NA | 0.201367986 | 0.260589 |
| Monteverdia floribunda | NA | 0.107203638 | 0.260589 |
| Moquiniastrum polymorphum | 0.0003625 | 0.0003625 | 0.0003625 |
| Muellera campestris | 0.08 | 0.08 | 0.08 |
| Myrceugenia alpigena | NA | 0.35124965 | 0.260589 |
| Myrceugenia euosma | NA | 0.303997477 | 0.260589 |
| Myrceugenia foveolata | 0.011195833 | 0.011195833 | 0.011195833 |
| Myrceugenia glaucescens | NA | 0.312978748 | 0.260589 |
| Myrceugenia mesomischa | 0.012247368 | 0.012247368 | 0.012247368 |
| Myrceugenia miersiana | 0.01269 | 0.01269 | 0.01269 |
| Myrceugenia myrcioides | 0.029884906 | 0.029884906 | 0.029884906 |
| Myrceugenia ovalifolia | NA | 0.193662797 | 0.260589 |
| Myrceugenia ovata | NA | 0.232503918 | 0.260589 |
| Myrceugenia oxysepala | NA | 0.137822858 | 0.260589 |
| Myrceugenia regnelliana | NA | 0.075336356 | 0.260589 |
| Myrcia | 0.029 | 0.029 | 0.029 |
| Myrcia aethusa | NA | 0.128501638 | 0.260589 |
| Myrcia brasilensis | NA | 0.074655218 | 0.260589 |
| Myrcia catharinensis | NA | 0.068024289 | 0.260589 |
| Myrcia dichrophylla | NA | 0.158213312 | 0.260589 |
| Myrcia glabra | NA | 0.078159182 | 0.260589 |
| Myrcia guianensis | 0.02 | 0.02 | 0.02 |
| Myrcia hartwegiana | NA | 0.152022986 | 0.260589 |
| Myrcia hatschbachii | NA | 0.161287779 | 0.260589 |
| Myrcia lajeana | NA | 0.079275103 | 0.260589 |
| Myrcia oblongata | NA | 0.173179829 | 0.260589 |
| Myrcia oligantha | 0.0382625 | 0.0382625 | 0.0382625 |
| Myrcia palustris | 0.0179 | 0.0179 | 0.0179 |
| Myrcia pubipetala | NA | 0.138655254 | 0.260589 |
| Myrcia retorta | NA | 0.186604186 | 0.260589 |
| Myrcia richardiana | NA | 0.058743406 | 0.260589 |
| Myrcia selloi | 0.0165 | 0.0165 | 0.0165 |
| Myrcia spectabilis | 0.1046 | 0.1046 | 0.1046 |
| Myrcia splendens | 0.140410715 | 0.140410715 | 0.140410715 |
| Myrcia tijucensis | 0.38 | 0.38 | 0.38 |
| Myrcia undulata | NA | 0.08544927 | 0.260589 |
| Myrcianthes gigantea | NA | 0.277183364 | 0.260589 |
| Myrcianthes pungens | 0.31315 | 0.31315 | 0.31315 |
| Myrciaria cuspidata | NA | 0.152491687 | 0.260589 |
| Myrciaria delicatula | 0.047654545 | 0.047654545 | 0.047654545 |
| Myrciaria floribunda | 0.1415 | 0.1415 | 0.1415 |
| Myrciaria plinioides | 0.3096 | 0.3096 | 0.3096 |
| Myrocarpus frondosus | 0.0524 | 0.0524 | 0.0524 |
| Myrrhinium atropurpureum | 0.009055661 | 0.009055661 | 0.009055661 |
| Myrsine | 0.01 | 0.01 | 0.01 |
| Myrsine coriacea | 0.011963044 | 0.011963044 | 0.011963044 |
| Myrsine gardneriana | NA | 0.050231384 | 0.260589 |
| Myrsine guianensis | 0.026 | 0.026 | 0.026 |
| Myrsine hermogenesii | NA | 0.056769457 | 0.260589 |
| Myrsine loefgrenii | NA | 0.047574086 | 0.260589 |
| Myrsine lorentziana | 0.014933333 | 0.014933333 | 0.014933333 |
| Myrsine umbellata | 0.300425 | 0.300425 | 0.300425 |
| Nectandra grandiflora | 1.11 | 1.11 | 1.11 |
| Nectandra lanceolata | 0.52134 | 0.52134 | 0.52134 |
| Nectandra megapotamica | 0.40816327 | 0.40816327 | 0.40816327 |
| Nectandra membranacea | 0.1845 | 0.1845 | 0.1845 |
| Nectandra oppositifolia | 0.7692 | 0.7692 | 0.7692 |
| Nectandra puberula | NA | 0.564747397 | 0.260589 |
| Neomitranthes gemballae | NA | 0.107004206 | 0.260589 |
| Ocotea bicolor | 0.06903333 | 0.06903333 | 0.06903333 |
| Ocotea catharinensis | 0.83333333 | 0.83333333 | 0.83333333 |
| Ocotea corymbosa | 0.2597 | 0.2597 | 0.2597 |
| Ocotea diospyrifolia | 0.5556 | 0.5556 | 0.5556 |
| Ocotea elegans | 0.35545 | 0.35545 | 0.35545 |
| Ocotea indecora | 0.19 | 0.19 | 0.19 |
| Ocotea laxa | NA | 0.377036063 | 0.260589 |
| Ocotea mandioccana | NA | 0.445043264 | 0.260589 |
| Ocotea nectandrifolia | NA | 0.467436792 | 0.260589 |
| Ocotea odorifera | 0.3003 | 0.3003 | 0.3003 |
| Ocotea porosa | 0.54466 | 0.54466 | 0.54466 |
| Ocotea puberula | 0.177755357 | 0.177755357 | 0.177755357 |
| Ocotea pulchella | 0.0863 | 0.0863 | 0.0863 |
| Ocotea pulchra | NA | 0.466467197 | 0.260589 |
| Ocotea riedeliana | NA | 0.453976378 | 0.260589 |
| Ocotea silvestris | 0.235 | 0.235 | 0.235 |
| Oreopanax fulvus | 0.0281 | 0.0281 | 0.0281 |
| Ormosia arborea | 1.25 | 1.25 | 1.25 |
| Ouratea parviflora | NA | 0.477204815 | 0.260589 |
| Pachystroma longifolium | 0.7692 | 0.7692 | 0.7692 |
| Parapiptadenia rigida | 0.0299 | 0.0299 | 0.0299 |
| Pausandra morisiana | NA | 0.170298549 | 0.260589 |
| Pera glabrata | 0.0196 | 0.0196 | 0.0196 |
| Persea major | NA | 0.723334511 | 0.260589 |
| Persea willdenovii | NA | 0.698155502 | 0.260589 |
| Phytolacca dioica | 0.0056 | 0.0056 | 0.0056 |
| Picramnia parvifolia | NA | 0.365373899 | 0.260589 |
| Pilocarpus pennatifolius | 0.044930953 | 0.044930953 | 0.044930953 |
| Pimenta pseudocaryophyllus | 0.11 | 0.11 | 0.11 |
| Piptadenia gonoacantha | 0.0625 | 0.0625 | 0.0625 |
| Piptocarpha angustifolia | 0.0082333 | 0.0082333 | 0.0082333 |
| Piptocarpha axillaris | 0.00076759 | 0.00076759 | 0.00076759 |
| Pisonia ambigua | 0.1471 | 0.1471 | 0.1471 |
| Pisonia zapallo | 0.1471 | 0.1471 | 0.1471 |
| Plinia peruviana | 0.182857143 | 0.182857143 | 0.182857143 |
| Plinia pseudodichasiantha | NA | 0.697232181 | 0.260589 |
| Podocarpus lambertii | 0.01425 | 0.01425 | 0.01425 |
| Posoqueria latifolia | 0.432366667 | 0.432366667 | 0.432366667 |
| Pouteria venosa | NA | 0.216942395 | 0.260589 |
| Protium kleinii | 0.38 | 0.38 | 0.38 |
| Prunus myrtifolia | 0.08325 | 0.08325 | 0.08325 |
| Prunus subcoriacea | NA | 0.208816238 | 0.260589 |
| Pseudobombax grandiflorum | 0.117 | 0.117 | 0.117 |
| Psidium cattleianum | 0.0137 | 0.0137 | 0.0137 |
| Psidium longipetiolatum | NA | 0.614021682 | 0.260589 |
| Psidium myrtoides | 1.3699 | 1.3699 | 1.3699 |
| Psychotria carthagenensis | 0.007183333 | 0.007183333 | 0.007183333 |
| Psychotria suterella | 0.02504 | 0.02504 | 0.02504 |
| Psychotria vellosiana | 0.38 | 0.38 | 0.38 |
| Quillaja brasiliensis | 0.0038 | 0.0038 | 0.0038 |
| Randia ferox | 0.2 | 0.2 | 0.2 |
| Recordia reitzii | NA | 0.104629915 | 0.260589 |
| Rhamnus sphaerosperma | 0.018155556 | 0.018155556 | 0.018155556 |
| Rollinia rugulosa | NA | 0.414572913 | 0.260589 |
| Roupala brasiliensis | 0.02 | 0.02 | 0.02 |
| Roupala montana | 0.02 | 0.02 | 0.02 |
| Roupala rhombifolia | NA | 0.182530339 | 0.260589 |
| Rudgea jasminoides | NA | 0.237860862 | 0.260589 |
| Ruprechtia laxiflora | 0.05 | 0.05 | 0.05 |
| Sapium glandulosum | 0.037946154 | 0.037946154 | 0.037946154 |
| Schefflera angustissima | 0.0088 | 0.0088 | 0.0088 |
| Schefflera calva | 0.0364 | 0.0364 | 0.0364 |
| Schinus | 0.01 | 0.01 | 0.01 |
| Schinus lentiscifolius | NA | 0.115105419 | 0.260589 |
| Schinus terebinthifolius | 0.0274 | 0.0274 | 0.0274 |
| Schizolobium parahyba | NA | 0.250692676 | 0.260589 |
| Scutia buxifolia | 0.13 | 0.13 | 0.13 |
| Sebastiania brasiliensis | 0.021 | 0.021 | 0.021 |
| Sebastiania commersoniana | 0.0043 | 0.0043 | 0.0043 |
| Senna multijuga | 0.0128 | 0.0128 | 0.0128 |
| Siphoneugena reitzii | NA | 0.27594053 | 0.260589 |
| Sloanea guianensis | 0.2 | 0.2 | 0.2 |
| Sloanea hirsuta | NA | 0.218790289 | 0.260589 |
| Sloanea lasiocoma | NA | 0.224373453 | 0.260589 |
| Sloanea monosperma | NA | 0.241040364 | 0.260589 |
| Solanum | 0.003 | 0.003 | 0.003 |
| Solanum johannae | 0.001571557 | 0.001571557 | 0.001571557 |
| Solanum mauritianum | NA | 0.063251782 | 0.260589 |
| Solanum pabstii | NA | 0.054120813 | 0.260589 |
| Solanum pseudoquina | 0.0035 | 0.0035 | 0.0035 |
| Solanum sanctaecatharinae | 0.005070136 | 0.005070136 | 0.005070136 |
| Solanum variabile | NA | 0.04347113 | 0.260589 |
| Sorocea bonplandii | 0.159525 | 0.159525 | 0.159525 |
| Strychnos brasiliensis | 0.198603448 | 0.198603448 | 0.198603448 |
| Styrax acuminatus | NA | 0.136948005 | 0.260589 |
| Styrax leprosus | 0.125 | 0.125 | 0.125 |
| Syagrus romanzoffiana | 3.52 | 3.52 | 3.52 |
| Symphyopappus itatiayensis | NA | 0.154222822 | 0.260589 |
| Symplocos tenuifolia | NA | 0.152743969 | 0.260589 |
| Symplocos tetrandra | NA | 0.206625508 | 0.260589 |
| Symplocos uniflora | 0.109728571 | 0.109728571 | 0.109728571 |
| Tabernaemontana catharinensis | 0.0553 | 0.0553 | 0.0553 |
| Tetrorchidium rubrivenium | 0.0149 | 0.0149 | 0.0149 |
| Tibouchina sellowiana | 2.77778E-05 | 2.78E-05 | 2.77778E-05 |
| Trema micrantha | NA | 0.137399597 | 0.260589 |
| Trichilia claussenii | 0.4251 | 0.4251 | 0.4251 |
| Trichilia elegans | NA | 0.23525464 | 0.260589 |
| Trichilia lepidota | 0.3809 | 0.3809 | 0.3809 |
| Trichilia pallens | 0.147058824 | 0.147058824 | 0.147058824 |
| Urera baccifera | 0.0007 | 7.00E-04 | 0.0007 |
| Vernonanthura discolor | 0.00051642 | 0.00051642 | 0.00051642 |
| Vernonanthura puberula | NA | 0.116226637 | 0.260589 |
| Virola bicuhyba | 1.2957 | 1.2957 | 1.2957 |
| Vitex megapotamica | 0.2602 | 0.2602 | 0.2602 |
| Weinmannia humilis | NA | 0.220257727 | 0.260589 |
| Weinmannia paulliniifolia | 0.008 | 0.008 | 0.008 |
| Xylopia brasiliensis | 0.073 | 0.073 | 0.073 |
| Xylosma ciliatifolia | NA | 0.117419905 | 0.260589 |
| Xylosma pseudosalzmannii | 0.008310938 | 0.008310938 | 0.008310938 |
| Xylosma tweediana | NA | 0.108137571 | 0.260589 |
| Zanthoxylum astrigerum | NA | 0.103736251 | 0.260589 |
| Zanthoxylum caribaeum | NA | 0.104460086 | 0.260589 |
| Zanthoxylum fagara | 0.01622 | 0.01622 | 0.01622 |
| Zanthoxylum kleinii | NA | 0.117163439 | 0.260589 |
| Zanthoxylum rhoifolium | 0.0066 | 0.0066 | 0.0066 |
| Zanthoxylum riedelianum | NA | 0.09269589 | 0.260589 |
| Zollernia ilicifolia | 0.8333 | 0.8333 | 0.8333 |

**References**

Laliberté, E., Legendre, P., & Shipley, B. (2015). FD: measuring functional diversity from multiple traits, and other tools for functional ecology. *R Package*, Version 1.0-12.

Oksanen, A. J., Blanchet, F. G., Friendly, M., Kindt, R., Legendre, P., Mcglinn, D., … Szoecs, E. (2017). Package ‘ vegan ’, (January), 0–291. https://doi.org/ISBN 0-387-95457-0
